# Cell-Free DNA in Cerebrospinal Fluid Complements the Monitoring Value of Interleukin-10 in Newly Diagnosed Primary Central Nervous System Lymphoma

**DOI:** 10.1155/2023/5808731

**Published:** 2023-01-04

**Authors:** Wei Wang, DongMei Zou, Zhe Zhuang, Xiao Zhang, Li Zhang, JingJing Yin, CongWei Jia, Li Yuan, Hao Cai, Yan Zhang, Xuan Wang, MeiFen Zhang, DaoBin Zhou, Wei Zhang

**Affiliations:** ^1^Department of Hematology, Peking Union Medical College Hospital, Chinese Academy of Medical Sciences & Peking Union Medical College, Beijing 100730, China; ^2^Department of Hematology, Xuanwu Hospital, Capital Medical University, Beijing 100053, China; ^3^Department of Ophthalmology, Peking Union Medical College Hospital, Chinese Academy of Medical Sciences & Peking Union Medical College, Beijing 100730, China; ^4^Department of Clinical Laboratory, Peking Union Medical College Hospital, Chinese Academy of Medical Sciences & Peking Union Medical College, Beijing100730, China; ^5^Department of Hematology, Beijing Hospital, Beijing 100005, China; ^6^Department of Pathology, Peking Union Medical College Hospital, Chinese Academy of Medical Sciences & Peking Union Medical College, Beijing 100730, China

## Abstract

**Objectives:**

Primary central nervous system lymphoma (PCNSL) usually has a poor prognosis. Cerebrospinal fluid (CSF) interleukin (IL)-10 has shown diagnostic, prognostic, and monitoring value in our previous studies. Cell-free circulating tumor DNA can be detected in the CSF of refractory/relapse cases and has also shown monitoring value. However, information about its monitoring value in newly diagnosed PCNSL patients and comparisons of CSF IL-10 and CSF cell-free DNA (cfDNA) are scarce.

**Methods:**

We performed next-generation sequencing on paraffin-embedded tissue and the serial CSF cfDNA of 10 newly diagnosed PCNSL patients and on the baseline CSF cfDNA of 11 other central nervous system lymphoma patients. We also monitored the CSF IL-10 levels of the 10 newly diagnosed PCNSL patients.

**Results:**

In seven newly diagnosed PCNSL patients with sufficient baseline CSF cfDNA, six had ≥1 mutated genes in their CSF cfDNA. The most common were MYD88(4/7), PIM1(3/7), MLL2(3/7), and ETV6(2/7). We also identified multiple somatic mutations, most commonly in PIM1. MYD88L265P can be detected in both tumor tissue and CSF cfDNA. The genomic profiles of CFS cfDNA were similar in PCNSL and PIOL patients. Newly diagnosed PCNSL patients with persistently positive cfDNA and negative IL-10 progressed quickly, while those with negative cfDNA and negative IL-10 were in maintenance therapy for more than 18 months. Two patients without cfDNA had increased CSF IL-10 concentrations before disease relapse. These results indicate that negative CSF cfDNA predicts better results, and persistently positive CSF cfDNA predicts disease progression earlier than conventional magnetic resonance imaging.

**Conclusion:**

In conclusion, CSF cfDNA is a potential predictor of relapse and progression, which complements the monitoring value of CSF IL-10 in newly diagnosed PCNSL patients.

## 1. Introduction

Primary central nervous system lymphoma (PCNSL) is a rare subtype of primary extranodal non-Hodgkin lymphoma (NHL) and involves only the brain, leptomeninges, or spinal cord without evidence of systemic disease. The overall incidence rate was 0.47 per 100,000 person-years from 1980 to 2008 and is currently still increasing [1]. The pathology of the disease consists primarily of the nongerminal center B-cell (non-GCB) subtype of diffuse large B-cell lymphoma (DLBCL) [2]. The prognosis is usually poor, and early diagnosis is challenging. Biopsy and pathology after cranial magnetic resonance imaging (MRI) constitute the best method for PCNSL diagnosis, but there are frequent complications from the biopsy. Although the new technology of stereotactic brain biopsy reduces the risk of brain hemorrhage, [3] some patients may get a negative result because of glucocorticoid use or an insufficient amount of tissue [4].

Cerebrospinal fluid (CSF) via lumbar puncture is easy and quick, however, and typically causes only minor damage. Cytology and flow cytometry (FCM) analysis are important methods to detect lymphoma cells in CSF, both with specificity as high as 100% [5, 6]. However, the sensitivity of FCM analysis can also be as low as 13.8% and that of cytology is even lower, leading to a high false-negative rate [6]. FCM analysis is usually abnormal in patients with leptomeninges involvement, and in those with only brain parenchyma involvement, cytology and FCM analysis are of little diagnostic value [5].

Cell-free circulating tumor DNA (ctDNA) is fragmented DNA released by apoptotic tumor cells into the blood. This type of DNA can be detected in the plasma of DLBCL patients and has monitoring and prognosis value [7]. Similarly, ctDNA can also be detected in the plasma of central nervous system lymphoma patients, but with low sensitivity and little value in disease monitoring [8]. Grommes et al. detected ctDNA in the CSF of refractory/relapse PCNSL patients via next-generation sequencing (NGS) and found that the overlap with tumor tissue mutations was 11–37% and that it showed monitoring value [8, 9]. CSF ctDNA in patients with secondary central nervous system lymphoma (SCNSL) and PCNSL patients was also detected via droplet digital PCR (ddPCR) and showed diagnostic value as well [8, 10, 11]. However, few studies have performed NGS on the CSF ctDNA of newly diagnosed PCNSL patients.

We have explored the value of CSF interleukin (IL)-10 in PCNSL and found that CSF IL-10 ≥ 8.2 pg/ml can differentiate PCNSL well from other CNS tumors, with sensitivity and specialty of 95.5% and 96.1%, respectively [12]. Furthermore, we found that elevated CSF IL-10 predicts disease relapse [13], similar to other studies that have found that it has monitoring value [14]. No one has yet compared the monitoring value of CSF IL-10 to that of cfDNA in PCNSL follow-up until now, however, although many mutations in PCNSL can lead to nuclear factor kappa B (NF-*κ*B) pathway activation, [15] which in turn can promote IL-10 expression [16].

Here, we performed NGS on the CSF cell-free DNA (cfDNA) of newly diagnosed PCNSL patients in order to explore its diagnostic value, as well as to compare its monitoring value to that of CSF IL-10. In addition, we also compared the molecular profile of newly diagnosed PCNSL patients with that of other central nervous system lymphoma (CNSL) patients.

## 2. Methods

### 2.1. Patient Cohort and Sample Collection

The patient cohorts of this study included ten newly diagnosed PCNSL patients and eleven other CNSL patients, including three with refractory/recurrence (R/R) PCNSL, two with primary intraocular lymphoma (PIOL) with central nervous system (CNS) progression, two with systemic DLBCL relapsed in the CNS, one with newly diagnosed systemic DLBCL involving the CNS, and three with newly diagnosed PIOL. All patients were treated at Peking Union Medical College Hospital, Beijing, China.

The diagnosis and response assessments of PCNSL were made with reference to the guidelines formulated by the International Primary CNS Lymphoma Collaborative Group in 2005 [17]. Patients with positive human immunodeficiency virus or Epstein-Barr virus were excluded. Each patient signed a written informed consent document prior to the study. The study was approved by the ethical committee at our hospital and was performed in accordance with the Declaration of Helsinki (no. ZS-2040).

Patients with newly diagnosed PCNSL received lumber puncture at baseline, before each cycle of chemotherapy, every three months during maintenance therapy, and at the time of relapse or progression. Patients with other CNLs received lumber puncture before chemotherapy. One microliter of CSF was used to measure IL-10 concentration for four hours for the detection. The rest of the CSF samples (five to ten microliters) and matched buccal mucosa were stored at −80°C. Formalin-fixed paraffin-embedded (FFPE) tumor tissues were obtained and stored at room temperature prior to DNA extraction.

### 2.2. DNA Extraction and Library Preparation

A DNeasy Tissue Kit (Qiagen, USA) was used to extract DNA from the buccal mucosa (germline DNA) and tumor tissue (tumor DNA). Frozen CSF was first thawed, and then, a QIAamp Circulating Nucleic Acid Kit (Qiagen) was used to extract cfDNA from it. Next, we used a Qubit fluorometer, a Qubit dsDNA BR Assay Kit (Invitrogen, USA), and a LabChip GX Touch system (PerkinElmer, Shanghai, China) to estimate the DNA concentration and fragment length. After the ends were repaired, A-tailed and the adapters were ligated, double-stranded cfDNA fragments went on PCR cycles to generate enough fragments for hybridization to custom-designed biotinylated oligonucleotide probes (IDT, Coralville, IA, USA) covering 413 genes.

### 2.3. Capture Panel Design, Sequencing, and Processing

Sequencing was performed using a GeneSeq-2000 (GenePlus-Suzhou, Suzhou, China) with a read length of PE100 and a depth of 500–1,000× [18]. Ninety genes related to lymphoma were analyzed (Supplementary Table S1). NCrealSeq (version 1.2.0, GenePlus-Suzhou) and NCfilter (version 2.0.0, GenePlus-Suzhou) were used to remove terminal adaptor sequences and low-quality reads separately from the raw data of paired samples, and the remaining reads were mapped to the reference human genome (hg19) using a Burrows–Wheeler Aligner (BWA, version 0.7.15-r1140) tool. Picard's Mark Duplicates tool (version 2.6.0) was used to identify duplicate reads of a normal sample. Finally, realSeq was used to identify duplicate reads of cancer samples, and Picard tools (version 2.6.0) was used to mark the normal samples.

### 2.4. Sequence Data Analysis

Somatic insertions/deletions (indels) and single nucleotide variants (SNV) were detected by comparing tumor-normal pairs with TNscope (version 201808) and realDcaller (version 1.7.1, GenePlus-Suzhou). NChot (version 2.7.2, GenePlus-Suzhou) was used to merge the results of this analysis, and NCanno (version 1.1.3, GenePlus-Suzhou) was used to annotate them to multiple public databases. Next, NCSV (0.2.3, GenePlus-Suzhou) was used to identify split-read and discordant-read pairs in order to identify SVs. All candidate variants were manually checked using the integrative genomics viewer browser [19].

### 2.5. CSF IL-10 Detection

Fresh CSF samples were used to detect IL-10 concentrations by electrochemiluminescence immunoassay as previously described in earlier work [12, 13]. The detection range of IL-10 was 5.0 to 1000.0 pg/mL.

### 2.6. Statistical Methods

The results of the sequencing were summarized by RStudio and WPS Office.

## 3. Results

### 3.1. Clinicopathological Characteristics of the Ten Newly Diagnosed PCNSL Patients

The median age of the ten newly diagnosed PCNSL patients was 59 (24–67) years, and most (70%) were male. The histology results were B-NHL for three patients and DLBCL for seven patients (Table 1, Figure S1). CSF IL-10 levels were elevated in 89% (8/9) of patients. Two patients had eye involvement, and all patients were from a phase Ib/II single-arm and single-center clinical trial of a rituximab, lenalidomide, and high-dose methotrexate regimen followed by lenalidomide maintenance (no. NCT04120350).

### 3.2. Gene Mutations in CSF cfDNA and FFPE Tumor DNA in a Newly Diagnosed PCNSL Patient

Only one patient had his FFPE tumor DNA sequenced, others lacked tissue or sufficient FFPE tumor DNA. We detected five mutated genes with low VAFs in the tumor tissue, including MYD88^L265P^, TNFRSF14^A32V^, ETV6 (multiple somatic mutations), and CCND3^S259A^. Of these, MYD88^L265P^ and TNFRSF14 were also detected in the CSF cfDNA, with similar VAFs (Supplementary Table S2).

### 3.3. Pretreatment CSF Genomics of the Newly Diagnosed PCNSL Patients

Baseline CSF cfDNA was sufficient for NGS in seven patients with newly diagnosed PCNSL, three with R/R PCNSL and seven with other CNSL (two with PIOL with CNS progression, one with newly diagnosed SCNSL, one with a history of systemic DLBCL and CNS relapse, and three with newly diagnosed PIOL). Three newly diagnosed PCNSL patients had insufficient CSF cfDNA. Overall, the newly diagnosed patients had higher VAF results than the relapsed patients (Supplementary Table S3).

**Figure 1:**
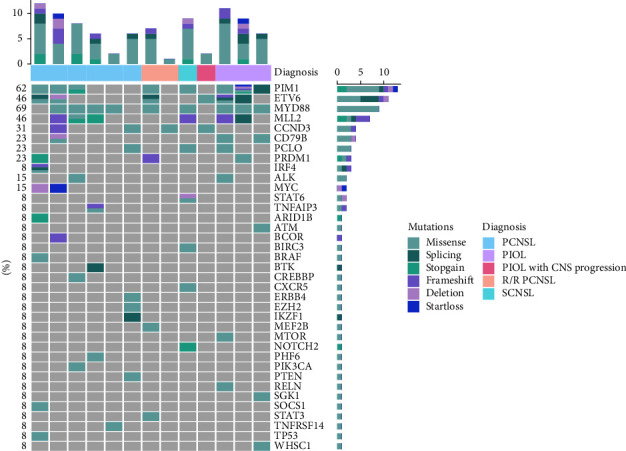
CSF cfDNA genomics of PCNSL and other CNSL patients at baseline.

**Figure 2:**
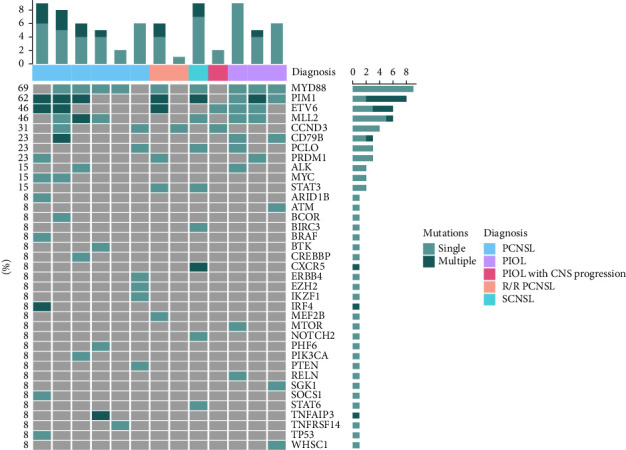
Single and multiple somatic mutations of CSF cfDNA at baseline.

Six newly diagnosed PCNSL patients had more than one mutated gene in their CSF cfDNA, and one had a negative result (Figure 1). Four patients had MYD88 mutation, with three having MYD88^L265P^ and one having MYD88^P258L^. One patient had CD79B mutation (multiple somatic mutations), and this patient also had MYD88^L265P^ mutation. Other common mutations included PIM1, ETV6, MLL2, and MYC. Four patients had B-cell receptor (BCR) pathway-associated mutations (Table 2). None of the patients had a CARD11 mutation. We also identified multiple somatic mutations from CSF cfDNA, and we observed that PIM1 (100%) and ETV6 (33%) were the most common mutation genes by NGS (Figure 2).

### 3.4. Comparison of the Genomic Profiles of CSF cfDNA between PCNSL and Other CNSL Patients

In three R/R PCNSL and other CNSL patients, the most common mutations were MYD88(57%), PIM1(43%), MLL2(43%), and ETV6(29%), similar to those of the newly diagnosed PCNSL patients. In the PIOL patients, CD79B, ETV6, PIM1, and MYD88 mutations were more common than in the PCNSL patients (Figure 1). In three R/R PCNSL and other CNSL patients, multiple somatic mutations were also detected, most commonly in PIM1, similar to the PCNSL patients (Figure 2).

### 3.5. CSF cfDNA Can Complement the Monitoring Value of CSF IL-10 in Newly Diagnosed PCNSL Patients

IL-10 detection and cfDNA NGS were performed on serial CSF in five newly diagnosed PCNSL patients with mutations in baseline CSF cfDNA. The VAFs of the CSF cfDNA decreased slowly, but CSF IL-10 became negative soon after chemotherapy. The other five newly diagnosed PCNSL patients only had IL-10 detection on serial CSF samples.

**Figure 3:**
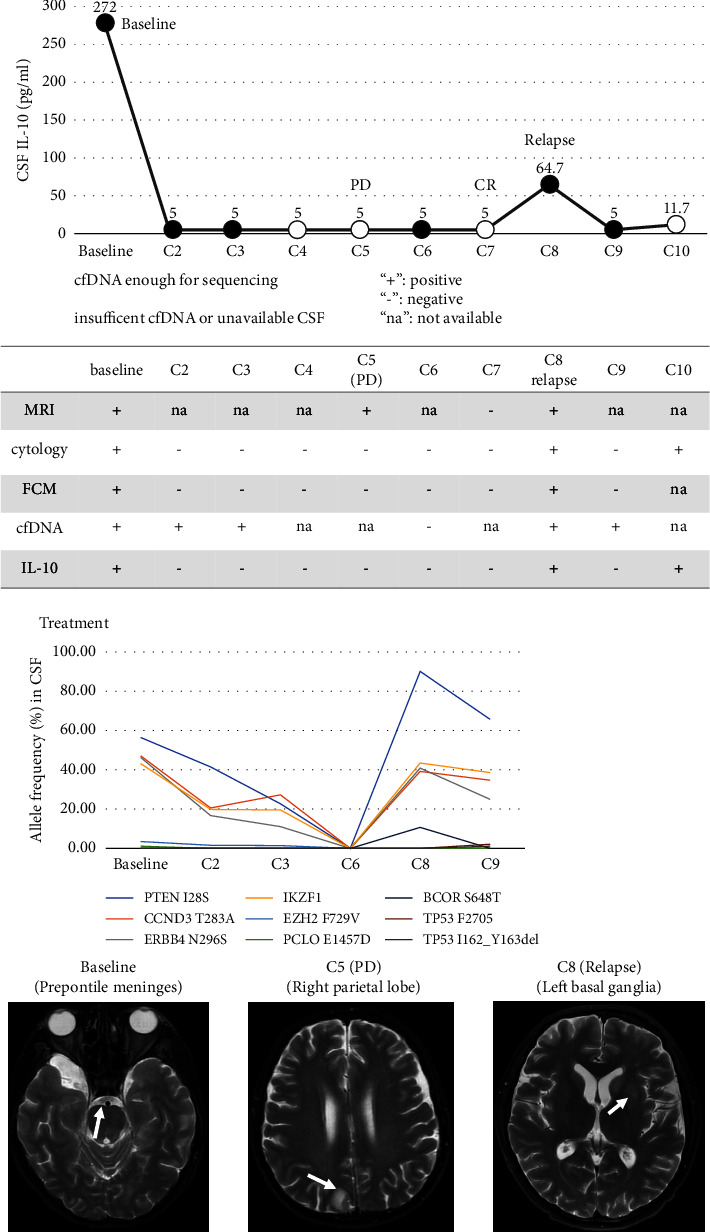
Disease monitoring through CSF cfDNA, CSF IL-10, and MRI of patient one. (a) CSF cfDNA, CSF IL-10, cytology, FCM, and MRI, (b) dynamic monitoring of cfDNA VAF, and (c) MRI. The cut-off value of CSF IL-10 was 9.1 pg/ml. White arrow: the tumor lesion. cfDNA: cell-free DNA, CSF: cerebrospinal fluid, FCM: flow cytometry, IL: interleukin, and MRI: magnetic resonance imaging.

Patient one was diagnosed by CSF FCM. His CSF IL-10 was 272.0 pg/ml at baseline (Figure 3), and mutations were detected in his CSF cfDNA, with a maximum VAF of 56.50%. CSF IL-10, FCM, and cytology became negative quickly after chemotherapy, but the VAFs of the CSF cfDNA mutation diminished slowly. After four cycles of chemotherapy, the disease progressed in MRI, when CSF IL-10 was still negative. After one cycle of second-line chemotherapy, the lesions disappeared and the CSF cfDNA was negative. However, the patient soon relapsed, CSF IL-10 increased to 64.5 pg/ml, and cfDNA became positive, with a maximum VAF of 90.10%. FCM and cytology were also positive for this patient. After the third-line therapy of PD-1 inhibitor (tislelizumab) and ibrutinib, IL-10, FCM, and cytology became negative again, but cfDNA was persistently positive. Patient one died two months later because of the disease's relapse.

**Figure 4:**
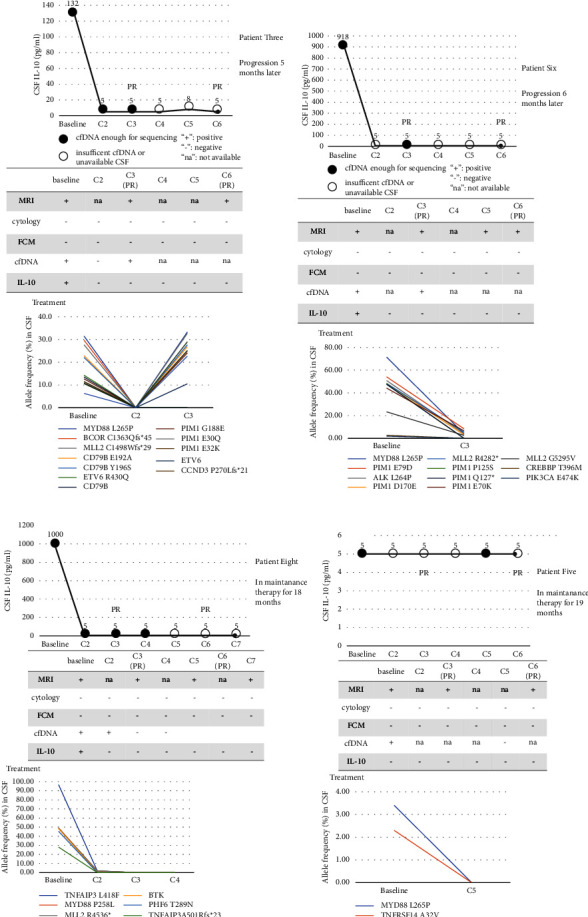
Disease monitoring through CSF cfDNA and CSF IL-10 of other newly diagnosed PCNSL patients. (a) Patient three, (b) patient six, (c) patient eight, and (d) patient five. The cut-off value of CSF IL-10 was 9.1 pg/ml. cfDNA: cell-free DNA, CSF: cerebrospinal fluid, FCM: flow cytometry, IL: interleukin, and MRI: magnetic resonance imaging.

For the other two patients (patient three and patient six) who were achieving PR and continuing maintenance therapy, although CSF IL-10 became negative quickly (Figures 4(a) and 4(b)), CSF cfDNA mutations could still be detected in their CSF. With these two patients, the disease had progressed after five and six months of maintenance therapy, respectively. However, IL-10 was negative and cfDNA was insufficient for NGS in patient six when the disease progressed, and CSF was unavailable in patient three. Patient three received pemetrexed and temozolomide as salvage therapy, and the disease progressed after four cycles. He then received a PD-1 inhibitor (camrelizumab) in a dosage of 200 mg every two weeks, and the disease progressed again after two doses. Patient six was receiving orelabrutinib and PD-1 inhibitor (sintilimab) as salvage therapy at the time of this writing.

**Figure 5:**
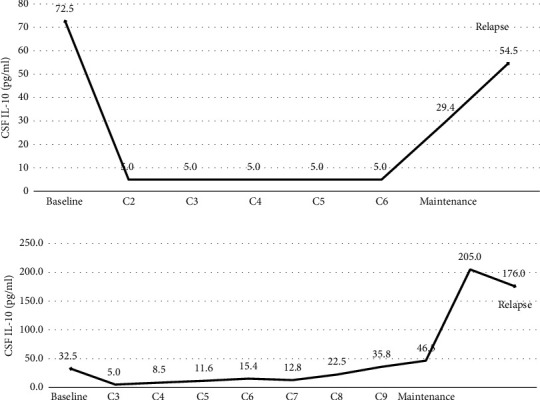
Disease monitoring through CSF IL-10 of two newly diagnosed PCNSL patients without baseline cfDNA. (a) Patient two and (b) patient ten.

Patients eight (Figure 4(c)) and five (Figure 4(d)) with persistently negative cfDNA and IL-10 after chemotherapy were still in a maintenance therapy period for 18 months and 19 months, respectively, without progression. In four patients without baseline CSF cfDNA, two were still in a state of remission and the other two had relapsed (Figure 5), with increased CSF IL-10 concentrations 154 and 444 days before relapse, respectively. In patients without leptomeninges involvement, FCM and cytology were negative at all time points.

## 4. Discussion

The results of this study, which are based on dynamic monitoring of CSF cfDNA and IL-10 concentrations, are consistent with those of previous studies demonstrating IL-10 and cfDNA as biomarkers for PCNSL. Most importantly, we compared the monitoring value of CSF cfDNA with IL-10 and found that CSF cfDNA can complement the monitoring value of IL-10 in newly diagnosed PCNSL patients. In addition, we found that elevated CSF IL-10 concentrations predicted disease relapse 154 and 444 days before disease relapse in two patients. This is similar to our previous study's finding that elevated IL-10 concentrations predicted relapse a median of 67 days (28–402 days) ahead of time in 10/16 patients [13]. CSF cfDNA also exhibited a monitoring role as VAF levels are reduced after chemotherapy and became positive or increased when disease relapse or progression occurred. CSF cfDNA was also often positive when patients were still in a PR state, though CSF IL-10 was negative. Patients with negative cfDNA after chemotherapy were still in maintenance therapy for more than 18 months, but those with persistently positive cfDNA suffered disease relapse or progression quickly after finishing chemotherapy. This suggests that CSF cfDNA is a potential predictor of relapse and progression, which complements the monitoring value of CSF IL-10 in newly diagnosed PCNSL patients. However, whether CSF cfDNA or IL-10 is more sensitive in predicting disease progression remains unknown due to our small cohort.

MYD88, CD79B, and other NF-*κ*B-pathway-related mutations promote IL-10 expression by lymphoma cells [15, 16, 20], and IL-10 has also been found to express in CD68^+^ and CD163^+^ tumor-associated macrophages by double immunostaining analysis [21]. Thus, we speculate that CSF cfDNA and IL-10 are generated in different ways, the former from tumor cells and the latter from both tumor cells and the tumor microenvironment. This may explain the inconsistent changes of these markers in different patients, and it also suggests that the combination of the two biomarkers may better monitor PCNSL than either of them alone.

In our study, the most frequent mutations detected in CSF were MYD88 (57%), PIM1 (43%), MLL2 (43%), and ETV6 (29%), similar to the genomics of PCNSL [22]. Multiple somatic mutations were most common in PIM1, which is similar to the results in Fukumura et al.'s study [22]. CD79B and MYD88 mutations are the hallmark of PCNSL [15], and in this study, although only one tumor tissue sample was available, MYD88^L265P^ can be detected in both CSF cfDNA and tumor tissue, with similar VAFs, so this suggests that CSF cfDNA reflects the genomic information of a tumor to some degree and might play a role in the diagnosis of CNSL. All seven of the newly diagnosed PCNSL patients had MYDL265P in their CSF cfDNA or baseline IL-10 ≥ 8.2 pg/ml, the latter of which has been shown to help diagnose CNSL [12]. Hence, the combination of CSF cfDNA and IL-10 may do an even better job of diagnosing CNSL, a result we have already found with PIOL [21].

An uncommon subset of PCNSL, PIOL, is involved only in vitreous, retinal, or optic tissues and can easily to relapse into the brain. Indeed, most PIOL patients die of brain relapse or progression [23]. Like PCNSL, most PIOL originates from B cells. Studies have found that MYD88^L265P^ and CD79B^Y196^ are positive in most PIOL biopsy specimens, similar to PCNSL [24]. In this study, MYD88, CD79B, PIM1, and MLL2 mutations were each detected in the CSF DNA of both newly diagnosed PIOL and PCNSL patients. These similar genetic characteristics suggest that PIOL and PCNSL may share the same biological characteristics and pathogenesis.

This study is also not without its limitations. First, the number of PCNSL patients was too small to compare the monitoring value of CSF cfDNA and IL-10 directly. A bigger cohort is needed to accomplish this in the future. Second, for some patients, CSF cfDNA was insufficient for NGS. A more sensitive detection method, such as ddPCR, is necessary, and the combination of NGS and ddPCR needs to be further studied as well. In addition, frozen CSF samples may affect the results of NGS, and perhaps, a better result can be achieved with fresh CSF [25]. We would better perform NGS on fresh CSF samples in the future.

## 5. Conclusions

Taken together, the results in this study suggested that CSF cfDNA can be a potential monitoring marker for PCNSL patients. However, the potential role of cfDNA as a surrogate marker for relapse and therapeutic response in PCNSL, and the comparison of CSF cfDNA and IL-10 need to be validated in a much larger cohort. We also found that CSF cfDNA could reflect the genomics of the tumor tissue of PCNSL patients, thus helping with diagnosis. Furthermore, the genomic profiles of CFS cfDNA were similar in PCNSL and PIOL patients.

## Figures and Tables

**Table 1:** Clinico-pathological characteristics of the ten newly diagnosed primary nervous system lymphoma patients.

Patient	Age	Gender	IELSG	Tumor location	Histology results	CD20	CD19	CD10	Bcl6	Bcl2	C-myc	Mum1	P53	Ki-67	EBER ISH	Flow cytometry	Surgery	CSF IL-10 (pg/ml)
1	48	M	3	Leptomeninges and nerve roots	B-NHL	+	+	+	N/A	N/A	N/A	N/A	N/A	60%	N/A	B-NHL^*∗*^	N/A	272.0
2	67	M	3	Eyes, ventricular system, right frontal lobe, and left basal ganglia	B-NHL	+	+	−	N/A	N/A	N/A	N/A	N/A	50%	N/A	B-NHL^#^	Rescetion^$^	72.5
3	62	M	3	Left corpus callosum and left cerebellum	DLBCL, GCB	+	N/A	+	+	+	−	+	−	70%	N/A	−	Biopsy	132.0
4	64	M	4	Left frontal lobe, left basal ganglia, and corpus callosum	DLBCL, non-GCB	+	N/A	−	+	+	−	+	+	80%	N/A	−	Rescetion	72.0
5	61	M	2	Sellar region and cavernous sinus	DLBCL, GCB	+	N/A	−	+	+	−	−	+	70%	−	−	Biopsy	5.0
6	54	F	1	Left basal ganglia, left cerebellum, and hypothalamus	DLBCL, GCB	+	N/A	+	+	+	+	+	+	90%	−	−	Biopsy	918.0
7	49	M	1	Left frontal lobe	DLBCL, non-GCB	+	N/A	−	−	+	+	−	−	85%	−	−	Rescetion	N/A
8	60	F	3	Bilateral frontal lobes	DLBCL, GCB	+	N/A	+	+	+	+	N/A	−	85%	−	−	Rescetion	1000.0
9	24	F	1	Cerebellum	DLBCL, GCB	+	+	+	−	−	−	−	N/A	N/A	−	−	Rescetion	95.4
10	58	M	1	Eyes and precentral gyrus	B-NHL	N/A	+	−	N/A	N/A	N/A	N/A	N/A	80%	N/A	B-NHL^#^	Rescetion^$^	32.5

M: male, F: female, IELSG: international extranodal lymphoma study group, DLBCL: diffused large B cell lymphoma, GCB: germinal center B cell, non-GCB: nongerminal center B cell, B-NHL: B cell nonhodgkin lymphoma, EBER ISH: the in situ hybridization of EBV-encoded RNA, CSF: cerebrospinal fluid, IL: interleukin, ^*∗*^: flow cytometry of cerebrospinal fluid, #: flow cytometry of vitreous fluid, $: resection of vitreous body, −: negative, and +: positive.

**Table 2:** Mutations in BCR pathway members in baseline CSF cfDNA of PCNSL patients.

ID	COO subtype	Best response	MYD88	CD79B	CARD11	BTK	TNFAIP3
P1	N/A	CR	WT	WT	WT	WT	WT
P3	GCB	PR	L265P	E192A/Y196S	WT	WT	WT
P4	Non-GCB	PR	WT	WT	WT	WT	WT
P5	GCB	PR	L265P	WT	WT	WT	WT
P6	GCB	PR	L265P	WT	WT	WT	WT
P8	GCB	PR	P258L	WT	WT	Splice	A501Rfs*∗*23
P10	N/A	CR	WT	WT	WT	WT	WT

COO: cell of origin, GCB: germinal center B cell, non-GCB: nongerminal center B cell, N/A: not available, and WT: wild-type.

## Data Availability

The data generated or analyzed during this study are included in this published article and its supplementary information files; others are not publicly available due to the large original file and the privacy of patients but are available from the corresponding author upon reasonable request.

## Abbreviations

BCR: B-cell receptor
cfDNA: Cell-free DNA
CNS: Central nervous system
CNSL: Central nervous system lymphoma
CSF: Cerebrospinal fluid
ctDNA: Cell-free circulating tumor DNA
ddPCR: Droplet digital PCR
DLBCL: Diffused large B-cell lymphoma
FCM: Flow cytometry
FFPE: Formalin-fixed paraffin-embedded
GCB: Germinal center B-cell
IL: Interleukin
MRI: Magnetic resonance imaging
NF-*κ*B: Nuclear factor kappa B
NGS: Next generation sequencing
NHL: Non-Hodgkin lymphoma
Non-GCB: Nongerminal center B cell
PCNSL: Primary central nervous system lymphoma
PIOL: Primary intraocular lymphoma
R/R: Refractory/recurrence
SCNSL: Secondary central nervous system lymphoma
VAF: Variant allele frequency.

## Supplementary Materials

Supplementary Materials Supplementary Figure S1: immunohistochemistry staining results of patient four. Supplementary Table S1: genes included in the panel.mmc1. Supplementary Table S2: the sequencing results of the brain tumor tissue and baseline CSF cfDNA in a newly diagnosed PCNSL patient. Supplementary Table S3: detailed information of gene mutations detected in CSF cfDNA of all the patients.
