# Supplementary material for: Cell-Free DNA in Cerebrospinal Fluid Complements the Monitoring Value of Interleukin-10 in Newly Diagnosed Primary Central Nervous System Lymphoma
Source: J Oncol. 2023 Jan 4;2023:5808731. doi: 10.1155/2023/5808731 (PMC9836788; doi:10.1155/2023/5808731)
Supplement: Supplementary Materials — Supplementary Figure S1: immunohistochemistry staining results of patient four. Supplementary Table S1: genes included in the panel.mmc1. Supplementary Table S2: the sequencing results of the brain tumor tissue and baseline CSF cfDNA in a newly diagnosed PCNSL patient. Supplementary Table S3: detailed information of gene mutations detected in CSF cfDNA of all the patients. [file 5808731.f1.zip › Additional Tables - 1 (2).pdf]

Additional Table S1

Genes included in the panel

**90 genes**					
ALK	ARID1A	ARID1B	ATM	B2M	BCL2
BCL6	BCOR	BIRC3	BRAF	BTK	CARD11
CCND1	CCND2	CCND3	CD274(PDL1)	CD28	CD58
CD79A	CD79B	CDKN2A	CDKN2B	CREBBP	CXCR4
CXCR5	DDX3X	DNMT3A	EP300	EPHA7	ERBB4
ETV6	EZH2	FAS	FBXW7	FGFR1	FOXO1
GATA3	GNA13	ID3	IDH2	IKZF1	IRF4
IRF8	ITK	JAK1	JAK2	JAK3	KIT
KLHL6	KMT2A(MLL)	KMT2D(MLL2)	KRAS	MAP2K1	EF2B
MTOR	MYC	MYD88	NOTCH1	NOTCH2	AX5
PCLO	PDCD1LG2(PDL	PDGFRB	PHF6	PIK3CA	IM1
PLCG2	PRDM1	PTEN	RB1	RELN	HOA
SETD2	SF3B1	SGK1	SMARCA	SOCS1	TAT3
STAT5B	STAT6	SYK	TCF3	TET2	NFAIP3
TNFRSF14	TP53	TP63	WHSC1	WT1	PO1

Additional Table S2

The sequencing results of the brain tumor tissue and baseline CSF cfDNA in a newly diagnosed PCNSL patient

Gene	HGVS	VAF	CSF VAF
MYD88	NM_002468.4; exon4;c.794T> C; p.L265P	2.70%	2.45%
ETV6	NM_001987:e xon1:c.33+1G	1.53%	0
PTEN	NM_00130471 8:exon6:c.43+1 G>A	1.05%	0
DNMT3A	NM_00132089 3:exon14:c.A1 748G:p.Y583C	0	1.11%

Additional Table S3

Detailed information of gene mutations detected in CSF cfDNA of all the patients

Number	Diagnosis	Gene.refGe	transcript	base change	AAChange.	Func.refGe	VAF
Patient 1	PCNSL	PTEN	NM_000314.4	c.83T>G	p.I28S	EX2	56.5%
Patient 1	PCNSL	CCND3	NM_001760.3	c.847A>G	p.T283A	EX5	47.1%
Patient 1	PCNSL	ERBB4	NM_005235.2	c.887A>G	p.N296S	EX8	46.3%
Patient 1	PCNSL	IKZF1	NM_006060.4	c.41-2A>G	.	IVS2	43.2%
Patient 1	PCNSL	EZH2	NM_004456.4	c.2185T>G	p.F729V	EX19	3.5%
Patient 1	PCNSL	PCLO	NM_033026.5	c.4371A>C	p.E1457D	EX5	1.1%
Patient 3	PCNSL	MYD88	NM_002468.4	c.794T>C	p.L265P	EX5	31.5%
Patient 3	PCNSL	BCOR	NM_001123385.	c.4085TG[2>1]	p.C1363Qfs	EX9	29.7%
Patient 3	PCNSL	MLL2	NM_003482.3	c.4492TG[2>1]	p.C1498Wf	EX16	27.7%
Patient 3	PCNSL	CD79B	NM_000626.2	c.575A>C	p.E192A	EX5	22.9%
Patient 3	PCNSL	CD79B	NM_000626.2	c.587A>C	p.Y196S	EX5	22.1%
Patient 3	PCNSL	ETV6	NM_001987.4	c.1289G>A	p.R430Q	EX8	14.3%
Patient 3	PCNSL	CD79B	NM_000626.2	c.72_118+23del	.	EX2-IVS2	13.5%
Patient 3	PCNSL	PIM1	NM_002648.3	c.563G>A	p.G188E	EX4	12.7%
Patient 3	PCNSL	PIM1	NM_002648.3	c.88G>C	p.E30Q	EX2	11.5%
Patient 3	PCNSL	PIM1	NM_002648.3	c.94G>A	p.E32K	EX2	11.4%
Patient 3	PCNSL	ETV6	NM_001987.4	c.14_33+8del CT	.	EX1-IVS1	10.8%
Patient 3	PCNSL	CCND3	NM_001760.3	c.807_846del CC	p.P270Lfs*2	EX5	10.6%
Patient 3	PCNSL	MYC	NM_002467.4	c.1C>G	p.0?	EX1	6.3%
Patient 4	PCNSL	TP53	NM_000546.5	c.524G>T	p.R175L	EX5	79.9%
Patient 4	PCNSL	PIM1	NM_002648.3	c.241C>T	p.P81S	EX4	79.2%
Patient 4	PCNSL	BRAF	NM_004333.4	c.1202C>G	p.T401S	EX10	63.8%
Patient 4	PCNSL	PIM1	NM_002648.3	c.4C>T	p.L2F	EX1	45.0%
Patient 4	PCNSL	PIM1	NM_002648.3	c.596C>T	p.T199M	EX4	44.1%
Patient 4	PCNSL	ETV6	NM_001987.4	c.463+1G>A	.	IVS4	42.3%
Patient 4	PCNSL	MYC	NM_002467.4	c.251_259delTCT	p.L84_S86d	EX2	41.5%
Patient 4	PCNSL	PIM1	NM_002648.3	c.286G>C	p.V96L	EX4	40.5%
Patient 4	PCNSL	ARID1B	NM_020732.3	c.349C>T	p.Q117*	EX1	39.4%
Patient 4	PCNSL	SOCS1	NM_003745.1	c.617C>T	p.S206F	EX2	35.3%
Patient 4	PCNSL	PIM1	NM_002648.3	c.73C>G	p.L25V	EX1	33.7%
Patient 4	PCNSL	PIM1	NM_002648.3	c.437G>A	p.S146N	EX4	33.1%
Patient 4	PCNSL	PIM1	NM_002648.3	c.403G>A	p.E135K	EX4	33.1%
Patient 4	PCNSL	PRDM1	NM_001198.3	c.201G>A	p.W67*	EX2	32.8%
Patient 4	PCNSL	ETV6	NM_001987.4	c.403C>T	p.H135Y	EX4	32.2%
Patient 4	PCNSL	PIM1	NM_002648.3	c.202C>T	p.H68Y	EX3	32.2%
Patient 4	PCNSL	IRF4	NM_002460.3	c.108G>T	p.K36N	EX2	30.8%
Patient 4	PCNSL	IRF4	NM_002460.3	c.-55-1G>C	.	IVS1	30.6%
Patient 4	PCNSL	IRF4	NM_002460.3	c.205delG	p.A69Rfs*3	EX2	27.3%
Patient 4	PCNSL	PIM1	NM_002648.3	c.550C>T	p.L184F	EX4	25.7%
Patient 4	PCNSL	IRF4	NM_002460.3	c.166C>T	p.H56Y	EX2	24.2%
Patient 4	PCNSL	IRF4	NM_002460.3	c.69G>C	p.K23N	EX2	22.4%
Patient 5	PCNSL	TNFRSF14	NM_003820.2	c.95C>T	p.A32V	EX2	3.4%
Patient 5	PCNSL	MYD88	NM_002468.4	c.794T>C	p.L265P	EX5	2.3%
Patient 6	PCNSL	MYD88	NM_002468.4	c.794T>C	p.L265P	EX5	71.5%
Patient 6	PCNSL	PIM1	NM_002648.3	c.237G>C	p.E79D	EX3	54.1%
Patient 6	PCNSL	ALK	NM_004304.4	c.791T>C	p.L264P	EX3	50.9%
Patient 6	PCNSL	PIM1	NM_002648.3	c.510C>G	p.D170E	EX4	48.4%
Patient 6	PCNSL	MLL2	NM_003482.3	c.12844C>T	p.R4282*	EX39	48.3%
Patient 6	PCNSL	PIM1	NM_002648.3	c.373C>T	p.P125S	EX4	47.7%
Patient 6	PCNSL	PIM1	NM_002648.3	c.379C>T	p.Q127*	EX4	47.5%
Patient 6	PCNSL	PIM1	NM_002648.3	c.208G>A	p.E70K	EX3	44.2%
Patient 6	PCNSL	MLL2	NM_003482.3	c.15884G>T	p.G5295V	EX49	23.3%
Patient 6	PCNSL	CREBBP	NM_004380.2	c.1187C>T	p.T396M	EX4	2.7%
Patient 6	PCNSL	PIK3CA	NM_006218.2	c.1420G>A	p.E474K	EX9	1.9%
Patient 8	PCNSL	TNFAIP3	NM_006290.3	c.1252C>T	p.L418F	EX7	96.7%

Patient 8	PCNSL	MYD88	NM_002468.4	c.773C>T	p.P258L	EX4	49.2%
Patient 8	PCNSL	MLL2	NM_003482.3	c.13606C>T	p.R4536*	EX40	48.7%
Patient 8	PCNSL	BTK	NM_000061.2	c.975-1G>T	.	IVS11	48.3%
Patient 8	PCNSL	PHF6	NM_032458.2	c.866C>A	p.T289N	EX9	45.4%
Patient 8	PCNSL	TNFAIP3	NM_006290.3	c.1501_1529d el	p.A501Rfs*:	EX7	28.0%
R1	R/R PCNSL	CCND3	NM_001760.3	c.775T>G	p.S259A	EX5	14.2%
R2	R/R PCNSL	MYD88	NM_002468.4	c.794T>C	p.L265P	EX5	9.0%
R2	R/R PCNSL	MEF2B	NM_001145785.	c.28C>T	p.R10C	EX2	7.2%
R2	R/R PCNSL	PIM1	NM_002648.3	c.193G>A	p.A65T	EX3	5.8%
R2	R/R PCNSL	STAT3	NM_003150.3	c.454C>T	p.R152W	EX5	5.8%
R2	R/R PCNSL	ETV6	NM_001987.4	c.33+1G>C	.	IVS1	5.3%
R2	R/R PCNSL	ETV6	NM_001987.4	c.17C>G	p.A6G	EX1	5.0%
R2	R/R PCNSL	PIM1	NM_002648.3	c.373C>T	p.P125S	EX4	4.2%
R2	R/R PCNSL	PIM1	NM_002648.3	c.83G>A	p.G28D	EX2	3.7%
R2	R/R PCNSL	PIM1	NM_002648.3	c.550C>T	p.L184F	EX4	3.2%
R2	R/R PCNSL	PRDM1	NM_001198.3	c.545_548dupTC	p.F184Lfs*6	EX4	3.1%
S1	SCNSL	MYD88	NM_002468.4	c.728G>A	p.S243N	EX4	99.0%
S1	SCNSL	STAT3	NM_003150.3	c.1959CAT[2>1]	p.I653[2>1]	EX21	52.6%
S1	SCNSL	MLL2	NM_003482.3	c.10198delC	p.Q3400Sfs	EX34	48.8%
S1	SCNSL	NOTCH2	NM_024408.3	c.7176T>G	p.Y2392*	EX34	36.9%
S1	SCNSL	PCLO	NM_033026.5	c.1459G>A	p.A487T	EX2	36.2%
S1	SCNSL	BIRC3	NM_182962.2	c.593G>A	p.G198E	EX3	19.7%
S1	SCNSL	PIM1	NM_002648.3	c.480C>G	p.N160K	EX4	3.5%
S1	SCNSL	PIM1	NM_002648.3	c.241C>G	p.P81A	EX4	3.3%
S1	SCNSL	STAT6	NM_003153.4	c.1678C>A	p.L560I	EX15	2.8%
S1	SCNSL	PIM1	NM_002648.3	c.94G>A	p.E32K	EX2	2.0%
S1	SCNSL	CXCR5	NM_001716.4	c.392T>G	p.V131G	EX2	1.9%
S1	SCNSL	CXCR5	NM_001716.4	c.335C>A	p.S112Y	EX2	1.4%
S1	SCNSL	PIM1	NM_002648.3	c.434G>A	p.R145H	EX4	1.3%
S1	SCNSL	PIM1	NM_002648.3	c.358C>G	p.L120V	EX4	1.1%
S1	SCNSL	PIM1	NM_002648.3	c.490C>T	p.L164F	EX4	1.1%
P1	PIOL with (	ETV6	NM_001987.4	c.1235G>A	p.G412E	EX7	10.60%
P1	PIOL with (	CCND3	NM_001760.3	c.775T>G	p.S259A	EX5	9.70%
O1	PIOL	RELN	NM_005045.3	c.6106G>A	p.A2036T	EX41	25.5%
O1	PIOL	MYD88	NM_002468.4	c.794T>C	p.L265P	EX5	23.4%
O1	PIOL	ALK	NM_004304.4	c.91T>C	p.S31P	EX1	18.8%
O1	PIOL	MLL2	NM_003482.3	c.5053A[6>7]	p.R1687Tfs	EX20	18.0%
O1	PIOL	ETV6	NM_001987.4	c.414CT[2>1]	p.S139Yfs*1	EX4	17.8%
O1	PIOL	ETV6	NM_001987.4	c.26G>A	p.S9N	EX1	16.4%
O1	PIOL	MTOR	NM_004958.3	c.635C>T	p.A212V	EX5	15.6%
O1	PIOL	CD79B	NM_000626.2	c.586T>A	p.Y196N	EX5	10.3%
O1	PIOL	PIM1	NM_002648.3	c.403G>A	p.E135K	EX4	8.8%
O1	PIOL	PCLO	NM_033026.5	c.4848C>A	p.S1616R	EX5	7.9%
O1	PIOL	ETV6	NM_001987.4	c.33+1G>A	.	IVS1	5.8%
O2	PIOL	CD79B	NM_000626.2	c.587A>G	p.Y196C	EX5	61.8%
O2	PIOL	WHSC1	NM_001042424.	c.332C>G	p.P111R	EX2	54.1%
O2	PIOL	PIM1	NM_002648.3	c.607+1G>A	.	IVS4	25.6%
O2	PIOL	ATM	NM_000051.3	c.9128A>G	p.K3043R	EX63	24.2%
O2	PIOL	MYD88	NM_002468.4	c.794T>C	p.L265P	EX5	21.3%
O2	PIOL	SGK1	NM_005627.3	c.202C>G	p.L68V	EX3	18.4%
O3	PIOL	ETV6	NM_001987.4	c.33+1G>A	.	IVS1	94.8%
O3	PIOL	PRDM1	NM_001198.3	c.291G>C	p.E97D	EX2	76.7%
O3	PIOL	MYD88	NM_002468.4	c.794T>C	p.L265P	EX5	62.1%
O3	PIOL	PIM1	NM_002648.3	c.143G>A	p.G48D	EX2	49.6%
O3	PIOL	MLL2	NM_003482.3	c.4418+1G>T	.	IVS15	45.4%
O3	PIOL	PIM1	NM_002648.3	c.-11_4delTGGA	p.0?	EX1	37.7%
O3	PIOL	PIM1	NM_002648.3	c.236_240+11de	.	EX3-IVS3	32.8%
O3	PIOL	PIM1	NM_002648.3	c.161C>G	p.S54*	EX2	10.5%

O3	PIOL	PIM1	NM_002648.3	c.265G>A	p.E89K	EX4	9.8%
O3	PIOL	PIM1	NM_002648.3	c.544C>T	p.L182F	EX4	9.3%
O3	PIOL	PIM1	NM_002648.3	c.94_101delGAG	p.E32Rfs*6[illegible]	EX2	5.3%
O3	PIOL	PIM1	NM_002648.3	c.291C>G	p.S97R	EX4	5.2%
O3	PIOL	PIM1	NM_002648.3	c.550C>T	p.L184F	EX4	3.8%
