# Supplementary figures and images for: Cell-Free DNA in Cerebrospinal Fluid Complements the Monitoring Value of Interleukin-10 in Newly Diagnosed Primary Central Nervous System Lymphoma
Source: J Oncol. 2023 Jan 4;2023:5808731. doi: 10.1155/2023/5808731 (PMC9836788; doi:10.1155/2023/5808731)

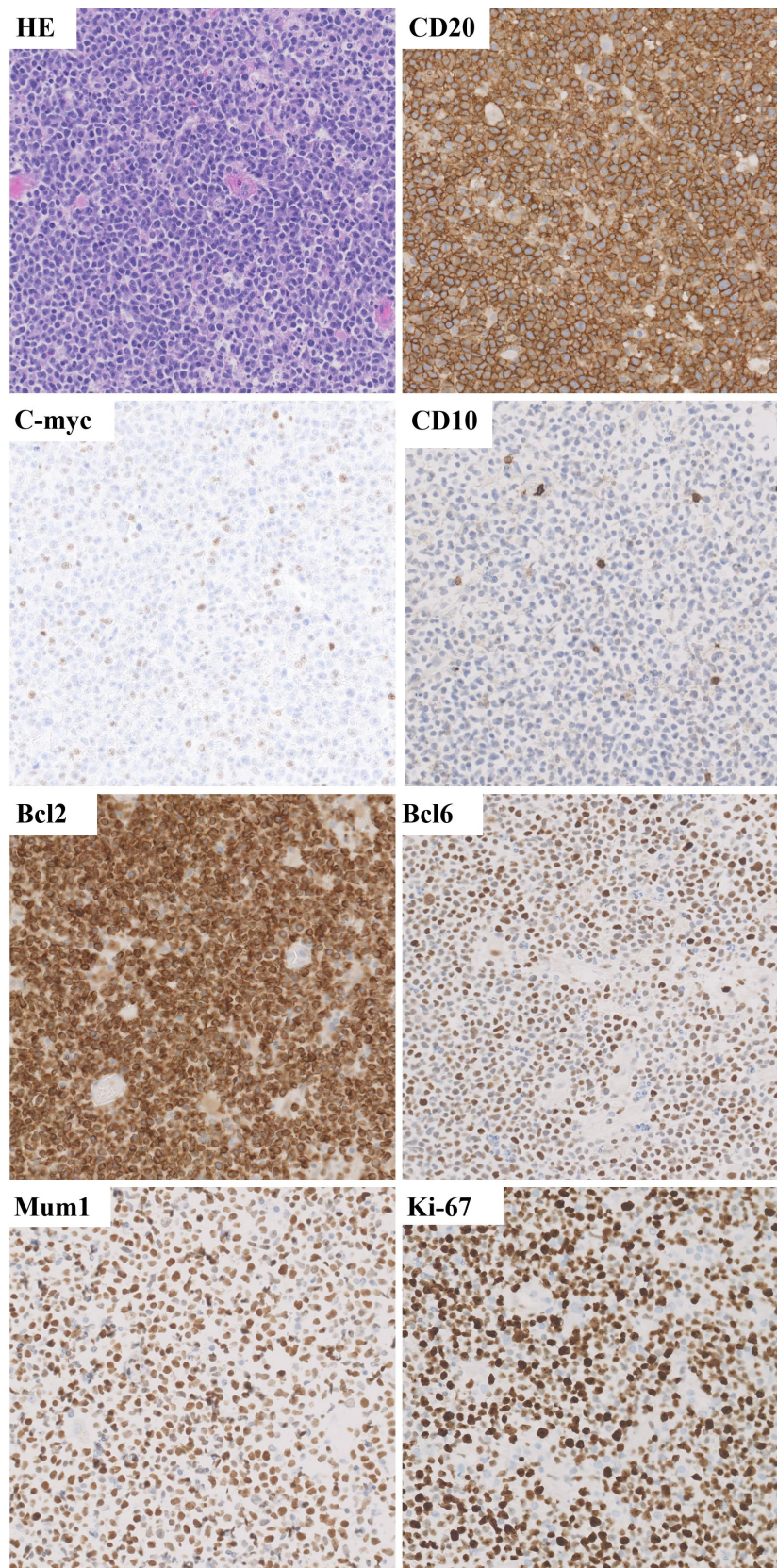

**Additional Figure S1.** Immunohistochemistry staining results of Patient Four.

Supplement: Supplementary Materials — Supplementary Figure S1: immunohistochemistry staining results of patient four. Supplementary Table S1: genes included in the panel.mmc1. Supplementary Table S2: the sequencing results of the brain tumor tissue and baseline CSF cfDNA in a newly diagnosed PCNSL patient. Supplementary Table S3: detailed information of gene mutations detected in CSF cfDNA of all the patients. [file 5808731.f1.zip › Additional Figure.pdf]
